# Talactoferrin Immunotherapy in Metastatic Renal Cell Carcinoma: A Case Series of Four Long-Term Survivors

**DOI:** 10.4021/jocmr499w

**Published:** 2011-02-12

**Authors:** Mark A. Lewis, Teresa G. Hayes

**Affiliations:** a Michael E. DeBakey Veterans Affairs Medical Center and Baylor College of Medicine, Houston, Texas, USA

## Abstract

Talactoferrin alfa (also known as recombinant human lactoferrin, rhLF) is a novel immunomodulatory protein that has previously demonstrated anti-tumor properties in animal models. Following a successful phase I trial, it was administered orally to patients with metastatic renal cell carcinoma (RCC) in a phase II trial conducted at the Michael E. DeBakey Veterans Affairs Medical Center in Houston, Texas, among other sites. We report a case series of 4 patients treated at our institution with very encouraging progression-free survivals, all exceeding 30 months, in order to suggest that this agent merits further study. These four patients with radiographically progressive metastatic RCC received single-agent oral talactoferrin in daily doses of 9 grams, given in cycles of 2 weeks on/2 weeks off, until evidence of toxicity or disease progression. Given the small sample size and the heterogenous tumor biology of RCC, tumor growth rate was used as a primary endpoint so that each patient could serve as their own control. The agent's effectiveness was then determined through radiographic tracking of the tumors before, during, and after treatment, with use of the Response Evaluation Criteria in Solid Tumors (RECIST) protocol to follow target lesions. The results showed that the drug was well tolerated, with no occurrence of talactoferrin-related grade 3 or 4 adverse events or laboratory anomalies by NCI-CTEP criteria. The four patients described in the case series demonstrated very encouraging progression-free survivals, all exceeding 30 months. We conclude that decreased tumor growth rate may correlate with increased progression-free survival. Talactoferrin is a promising, well-tolerated agent whose clinical benefits should be evaluated in a randomized phase III study with a placebo control arm.

**Keywords:**

Talactoferrin; Immunotherapy; Renal cell carcinoma; Metastatic

## Introduction

While radical nephrectomy offers the chance for cure in localized renal cell carcinoma, improving treatment for metastatic renal cell carcinoma remains a hotly pursued goal in clinical research. The predominant clear cell subtype of this malignancy is discouragingly resistant to chemotherapy [1]. Sharpened understanding of tumor neovascularity and dysregulation of hypoxia-inducible factor (HIF) [2] has led to promising results with therapies that target downstream vascular endothelial growth factor (VEGF) [3, 4]. However, even with increasing focus on antiangiogenic agents, efforts continue to develop effective and tolerable immunotherapy. The historical experience with interleukin-2 has shown clinical efficacy at high doses but its toxicity profile can limit the length and intensity of its use [5]. Ideally, newer immunotherapeutic strategies will provide effective antitumor responses without incurring as many side effects as IL-2.

Talactoferrin alfa (talactoferrin; TLF), a novel immunomodulatory agent, is a recombinant human lactoferrin (rhLF) purified from *Aspergillus*. Lactoferrin, an iron-binding glycoprotein, can be found throughout the body, first identified in breast milk [6] but also present in nasolacrimal secretions, bronchial and cervico-vaginal mucus, and inside the granules of phagocytes [7]. As a trigger for immunomodulation at the gut-associated lymphoid tissue (GALT) [8], and as a dendritic cell maturation agent [9, 10] lactoferrin possesses many potentially useful pharmacologic functions, including anti-tumor activity. Talactoferrin has demonstrated anti-tumor activity in multiple animal models of solid tumors [11]. A dose-escalation trial with expansion at the optimal dose has already been conducted at the Michael E. DeBakey Veterans Affairs Medical Center in Houston, Texas to investigate the safety, tolerability, and anti-tumor activity of oral talactoferrin in metastatic solid tumors, including renal cell carcinoma (RCC) [12].

In a phase IB trial [13] and a follow-on phase II study [14], 16 patients with metastatic RCC, all with documented tumor progression during the 9 months prior to enrollment, were treated at our institution with single-agent oral talactoferrin. Patients received oral rhLF in amounts of 4.5 grams or 9 grams per day administered in two divided doses. The drug was given in 14-day cycles interrupted by a 14-day rest interval, until evidence of toxicity or disease progression. One patient switched from the 4.5-gram dosage to the 9-gram dosage due to progression on the lower dose. Patients were requested to maintain a diary documenting consumption of the study drug and any side effects they experienced. Toxicities were graded according to National Cancer Institute common toxicity criteria [15]. Prior to entering he study, all patients had a complete history and physical examination, complete blood cell count, serum chemistries and electrolytes, and radiologic assessment of the measurable tumor mass. Subsequently, a research nurse and physician saw patients at regularly scheduled outpatient clinic visits. Complete blood count, serum electrolytes and chemistries were obtained at each visit. Tumor size progression was monitored through serial radiologic studies, performed at baseline, at 2 months, and approximately every 8 weeks thereafter. Target lesions were selected prospectively (prior to start of therapy) and followed using the Response Evaluation Criteria in Solid Tumors (RECIST) [16].

We report a case series of the longest-term survivors from these two trials, all with progression-free survival exceeding 30 months.

## Case Report

These four patients' clinical courses are synopsized in the following brief narratives and additional information, including age and MSKCC risk score from time of post-nephrectomy recurrence [17], can be found in Table 1.

**Table 1:** Additional Patients' Information

Sr. No.	1	2	3	4
Age at Entry	66	51	60	72
RhLF Dose	9 g/day	9 g/day	9 g/day	9 g/day
Histology	Clear cell	Clear cell	Clear, Spindle cell	Clear cell
Previous Therapy	nephrectomy	nephrectomy, IL-2, interferon-α	nephrectomy, IL2, interferon-α, methionine restriction	nephrectomy, Capecitabine, Gemcitabine, interferon-α, thalidomide
MSKCC Risk	Low	Low	Low	Low
Patient Status	Alive after 6 years	Alive after 5.5 years	Alive after 7.5 years	Died after 37 months
PFS (Months)	35	50	35	34

### Case 1

This patient underwent left radical nephrectomy in 1992 for grade II clear cell renal cell carcinoma with extension in the perinephric fat but no vascular invasion, no metastases to lymph nodes, and no positive surgical margins. In July 2004, he was found to have a heterogenous soft tissue mass in the right paratracheal area, extending to the right hilum, along with scattered lung nodules; biopsy during mediastinoscopy confirmed metastatic clear cell RCC. He was deemed to have unresectable disease and was enrolled in our talactoferrin trial. He began the 4.5 gm arm of the study in November 2004, at which time he was 66 years old. He developed progressive disease in June 2005 after his lesions grew 24% from baseline. In October 2005, he crossed over to the 9 gm arm of the study and his disease remained stable by RECIST criteria for 35 months until September 2008, when he developed a lytic lesion in his left femur. At this point his participation in the study ended and he was switched to sunitinib with focal radiotherapy for his bone lesion. He eventually underwent hip replacement due to progression of the bony disease. Subsequent treatments have included sorafenib, everolimus, and pazopanib.

### Case 2

This patient underwent left nephrectomy in 1995 for localized clear cell renal cell carcinoma. In December 2002, he was diagnosed with recurrent disease in the left renal surgical bed and with metastases to both lungs. After receiving IL-2 and interferon-α at another facility from February through June 2003, he initiated care at our hospital in September 2003, at which time he was continued on interferon-α. CT scans in March 2005 showed progressive disease and he was enrolled in the talactoferrin trial in June 2005. He remains on study, with progression-free survival not yet reached at 50 months. He remained on study until August 2009, when CT scans documented cancer progression. He received gamma knife treatment for a brain metastasis and systemic treatment with sunitinib followed by everolimus.

### Case 3

The patient underwent left nephrectomy in 1995 for an 18 cm clear cell/spindle cell renal cell carcinoma that invaded the capsule and extended into perinephric fat. In 1998 he developed left flank pain and was found to have biopsy-confirmed recurrent disease in the left renal surgical bed, as well as subcentimeter nodules in both lung bases. The patient was treated with IL-2 and interferon-α from 1998 to 1999 without response, then participated in an experimental methionine restriction diet from 1999 to 2000, during which he experienced disease progression. He was enrolled in the talactoferrin trial from July 2003 to June 2006 with stable disease. In June 2006, after 35 months of progression-free survival, he had disease progression and was changed to erlotinib plus bevacizumab. He did not tolerate sunitinib and was subsequently treated with sorafenib, temsirolimus, and then pazopanib.

### Case 4

**Figure 1.**
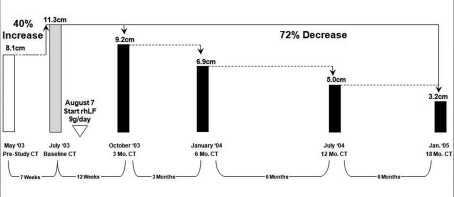
The regression of the tumor size (longest diameters) during the first 17 months of therapy in case 4.

**Figure 2.**
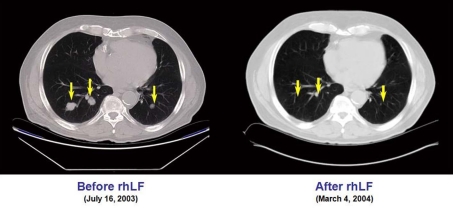
CT scans showing the reduction in size of the pulmonary metastases in case 4.

This patient underwent nephrectomy in 2002 for renal cell carcinoma. Lung metastases were diagnosed 6 weeks post-operatively and the patient was treated with capecitabine, interferon-α, gemcitabine, and thalidomide. After initial improvement, he progressed and entered the talactoferrin trial in August 2003. He had a partial response and then remained stable by RECIST criteria until July 2006. His tumor regression during the first 17 months of therapy is summarized graphically in Figure 1. Serial CT scans, seen in Figure 2, show the reduction in size of his pulmonary metastases. The patients cancer later progressed in the mediastinum and brain, and he expired in September, 2006.

## Discussion

These four promising examples must be interpreted within the appropriate context. First of all, these cases were selected for presentation from a larger pool of trial patients precisely because their progression-free survival was so encouraging. Secondly, their pre-study risk stratification, based upon time to post-nephrectomy recurrence, performance status, and lack of anemia, hypercalcemia, or elevated LDH, was uniformly low, although they had clearly documented tumor progression at the time of trial enrollment. Third, arrested tumor growth can occur as a part of the natural history of metastatic RCC. Under Gompertzian models, such time-dependent slowing occurs as the tumor growth curve reaches its plateau. As such, renal cell carcinomas under surveillance but not treatment can demonstrate a rate of volumetric expansion that diminishes with increasing size [18]. Nonetheless, in the three-pronged attack on renal cell carcinoma from the avenues of cell biology, angiogenesis, and immunology, well-tolerated immunomodulatory agents have been elusive. We suggest that the progression-free survivals of these patients merit further evaluation of oral talactoferrin to determine its true anti-neoplastic efficacy.

## Consents

Written informed consent was obtained from each patient for the educational use of his or her de-identified clinical information. Copies of the consents are available for review.
